# Time for change: a new training programme for morpho-molecular pathologists?

**DOI:** 10.1136/jclinpath-2017-204821

**Published:** 2017-11-07

**Authors:** David A Moore, Caroline A Young, Hayley T Morris, Karin A Oien, Jessica L Lee, J Louise Jones, Manuel Salto-Tellez

**Affiliations:** 1 Department of Cancer Studies, University of Leicester, Leicester, UK; 2 School of Medicine, St James’ University Hospital, Leeds, UK; 3 Institute of Cancer Sciences - Pathology, University of Glasgow, Glasgow, UK; 4 Strategy and Initiatives, National Cancer Research Institute, London, UK; 5 Centre for Tumour Biology, Barts Cancer Institute, Barts and the London School of Medicine and Dentistry, London, UK; 6 Northern Ireland Molecular Pathology Laboratory, Centre for Cancer Research and Cell Biology, Queen’s University Belfast, Belfast, UK

**Keywords:** histopathology, molecular pathology, morphology, cancer, diagnostics

## Abstract

The evolution of cellular pathology as a specialty has always been driven by technological developments and the clinical relevance of incorporating novel investigations into diagnostic practice. In recent years, the molecular characterisation of cancer has become of crucial relevance in patient treatment both for predictive testing and subclassification of certain tumours. Much of this has become possible due to the availability of next-generation sequencing technologies and the whole-genome sequencing of tumours is now being rolled out into clinical practice in England via the 100 000 Genome Project. The effective integration of cellular pathology reporting and genomic characterisation is crucial to ensure the morphological and genomic data are interpreted in the relevant context, though despite this, in many UK centres molecular testing is entirely detached from cellular pathology departments. The CM-Path initiative recognises there is a genomics knowledge and skills gap within cellular pathology that needs to be bridged through an upskilling of the current workforce and a redesign of pathology training. Bridging this gap will allow the development of an integrated ‘morphomolecular pathology’ specialty, which can maintain the relevance of cellular pathology at the centre of cancer patient management and allow the pathology community to continue to be a major influence in cancer discovery as well as playing a driving role in the delivery of precision medicine approaches. Here, several alternative models of pathology training, designed to address this challenge, are presented and appraised.

## Historical perspective of pathology: from an autopsy science to a clinical science and a pillar of research

The subject of pathology has been in evolution for over 2000 years, with progress made following waves of intellectual and technological advancement. Early pathological understanding arose from the clinical descriptions of infections, inflammation and tumours first described by Hippocrates. Renaissance-era physicians and anatomists developed this understanding by describing gross pathological appearances seen through human dissection, and later Thomas Hodgkin was the first physician to define pathology by the microscopic appearances in human tissue, including the disease that now bears his name.

The extensive work of Rudolf Virchow, by many regarded as the greatest figure in the history of pathology, in describing and categorising human disease by cellular appearances layed the basis for the development of pathology as both a clinical discipline and a modern science. In the late 19th and early 20th centuries, teachers in morbid anatomy were found in medical schools throughout Europe representing the early ‘academic pathologists’. Meanwhile, the clinical practice of ‘surgical pathology’ was beginning to be performed by physicians and surgeons who used microscopic findings for clinical diagnosis. By the mid-20th century, these roles were consolidated as histopathology became recognised as a medical diagnostic discipline in its own right, one based on light microscopy, and with a strong academic ethos.^1 2^ Many names have been applied to the discipline of identifying the shape and form of cells and their spatial arrangement in healthy and pathological tissue and cell samples: Tissue Pathology, Cellular Pathology, Anatomic Pathology, Histopathology, Surgical Pathology and Cytopathology. All of these will be synonymous from now for the purpose of this document.

## Twenty-first century pathology: a morphomolecular discipline?

In 1998, 45 years after the discovery of the DNA helix, the director of the National Cancer Institute challenged the scientific community to ‘harness the power of comprehensive molecular analysis technologies to make the classification of tumors vastly more informative’ and to change ‘the basis of tumor classification from morphological to molecular characteristics’.^3^ Almost 20 years later, to what extent has this challenge been met?

Traditional pathology training has taught us to recognise and distinguish a myriad of pathological entities based on their macroscopic and microscopic morphological appearances. Complex diagnoses are founded on years of collective experience and an element of subjectivity. This is in contrast with molecular diagnostic methods, which are heavily technology driven and produce objective and quantitative data. Immunohistochemistry (IHC) sits somewhere between the two.

While IHC has been adopted into routine practice and pathology training, in many institutions ‘traditional’ morphological pathology and ‘new molecular’ pathology laboratories exist in parallel, with little interaction. A notable exception to this is the work of the Specialist Integrated Haematological Malignancy Diagnostic Service (SIHMDS) in the UK. SIHMDS services combine elements of laboratory haematology, histopathology, flow cytometry, cytogenetics and molecular medicine prior to the production of a finalised integrated report, which permits internal validation and crosschecking. The development of this model has not been without its difficulties: centralisation of services, co-location of several laboratories and information technology and computing infrastructure. Cellular pathology has much to learn, both from the successes and difficulties that SIHMDS has experienced.^4–6^

## Established molecular testing based on tissues or cells

There are currently a number of tissue-based molecular tests used in routine diagnostic practice, although the exposure of individual pathologists will be dependent on their area of practice, as in some subspecialties, molecular diagnostics has progressed more rapidly than in others.^7^ Some examples of frequently used molecular tests are summarised in table 1.

**Table 1:** Established tissue-based molecular tests (adapted from Flynn *et al* 25)

Diagnostic	Therapeutic	Genetic
Lymphoma translocation detection	KRAS/NRAS mutation testing	MSI testing
Clonality testing	c-KIT and PDGFRA mutation analysis	MMR protein expression
Sarcoma translocation detection	BRAF mutation testing	
	EGFR mutation testing	
	ALK protein expression	
	EML4-ALK translocation detection	
	CNS tumours—multiple	
	ER, PR and HER2 protein expression	
	HER2 amplification	

CNS, central nervous system; MMR, mismatch repair; MSI, microsatellite instability.

As our understanding of cancer biology progresses and new technologies develop, extension of these single-gene tests or low-target tests has the potential to completely reshape the field of tissue-based and cell-based molecular testing.^8^ For example, next-generation sequencing is beginning to show its full potential for diagnostic and therapeutic applications, particularly for the reclassification of diagnosis, therapeutic decision-making beyond the classic organ-specific treatment options and the detection of standard-of-care mutations.

IHC already has an established role in the histopathological taxonomy of diseases, with disease-specific ‘patterns of immunohistochemical expression’ now a standard component of a pathologist’s diagnostic acumen. In recent decades, new uses have been postulated for some of these immunohistochemical biomarkers. For instance, the presence (or absence) of expression of a protein may be perceived as evidence of an inherited disease. In the field of cancer immunology, the assessment of markers of adaptive immunity (eg, CD3, CD4, CD8) and immune checkpoints (eg, PD-1, PD-L1, OX-40, LAG3, TIM3) show promise as prognostic and predictive indicators. Indeed, in the era of personalised medicine, immunohistochemical expression may dictate a certain therapeutic intervention.^9 10^

### Molecular classification of cancer

With the notable exceptions of haematological malignancies and more recently brain tumours, the mainstay of cancer classification is morphology. However, large-scale genomic profiling studies have demonstrated the level of tumour diversity at a genomic level: genomic and transcriptomic analysis of breast cancer identified 10 ‘integrative subtypes’ with prognostic and therapeutic implications,^11^ and similar approaches in other tumours have indicated important additive information from genomic profiling.^12–14^ Ongoing national whole-genome sequencing (WGS) initiatives, such as the 100,000 Genome Project in the UK and Genome British Columbia in Canada, are integrating high-throughput genomic profiling of tumours into routine healthcare services. With plans already underway for the introduction of WGS to clinical laboratories, the advent of truly integrated morphomolecular reporting for cancer is imminent. As well as requiring expertise in the validation, interpretation and integration of molecular and morphological analysis, the not inconsiderable challenges associated with these genomic projects have highlighted the critical importance of sample integrity. While morphology is relatively forgiving to the vagaries of variable tissue fixation, the integrity and validity of genomic analysis are heavily reliant on optimal and consistent sample handling. As the curators of tissue specimens, therefore, there is a strong case for ensuring that pathologists are equipped with the necessary knowledge and skills to be leaders in the development of protocols for tissue-based genomic analysis and to oversee their implementation and use in a diagnostic setting.^15^

### Improved biomarker discovery and validation

**Figure 1:**
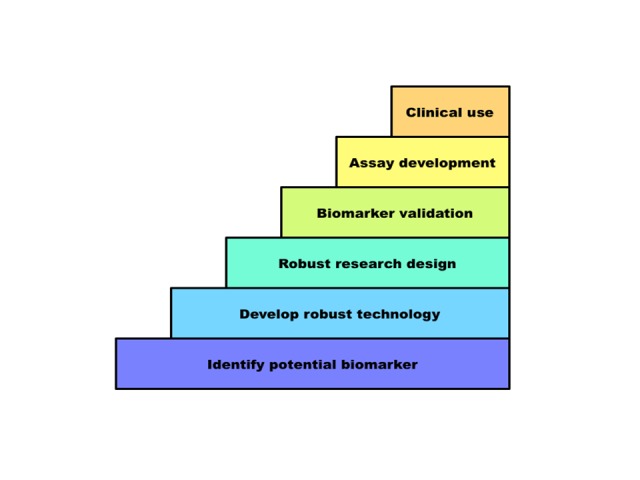
Steps to adoption of a new biomarker for clinical use. Frequent barriers to progressing through these steps include inadequate technology, flaws in research design including use of an irrelevant study population, underpowering and inappropriate statistical analysis and impracticality or expense of the assay (as discussed by Kern).^21^

High-throughput genomic profiling makes possible rapid and comprehensive analysis of cancer tissues even from small biopsy samples. Predictive (diagnostic) biomarkers can be used to match patients with targeted therapies. Prognostic markers can be integrated into existing clinical staging systems for improved patient stratification. However, in spite of the large number of credible biomarkers identified in basic research studies, relatively few have been validated and accepted for routine clinical use. The steps to the translation of biomarkers into clinical use are summarised in figure 1.^17^ As many of the barriers are related to the way we deal with samples, technology or study design, this reinforces the importance of upskilling the pathology workforce, most of whom will be involved in such studies to some degree, in the limitations of molecular pathology methods, optimum specimen processing and requirements for biomarker validation.

### Tumour heterogeneity and tumour evolution

The level of intratumour and intertumour heterogeneity^16 17^ and the existence of tumour evolution^18^ including divergence of metastatic lesions from the primary tumour, all present challenges to understanding the biology of and ultimately to the successful long-term management of cancer. A gap analysis conducted by the charity Breast Cancer Now^19^ identified the lack of knowledge of metastatic disease, its evolution and interaction with the metastatic microenvironment as critical blocks in the improvement of patient outcomes. The panel emphasised the importance of systematic longitudinal biobanking of matched primary and metastatic samples, but also highlighted the unrivalled value of the autopsy for tissue retrieval and global tumour analysis. Indeed, such initiatives in the UK and USA are leading to the re-emergence of the hospital autopsy to accelerate cancer research, and this places pathology at the forefront of discovery in this cutting-edge field.

## Embracing genomics: the expanding role of pathology

The impact of molecular technologies on patient assessment, diagnosis and management means the role of the pathologist will expand and evolve. Apart from traditional morphological assessment of tissues and cells, and accompanying molecular tests, pathology is at the interface between biomarker validation, biomarker adoption and diagnostics.

### Influencing therapeutic decisions

Pathologists have a key role in the integration of molecular medicine into therapeutic pathways through their influence in biomarker translation, clinical trials and clinical data systems.^20^

Much of the large failure rate in the lack of clinical applicability of biomarker studies is in the fact that validation and biomarker adoption are suboptimally executed.^21 22^ Knowledge of when a new or alternative biomarker is needed, how to design and power such studies, which technology should support their clinical delivery and how to take a biomarker through European Medicines Agency/Food and Drug Administration approval and National Health Service/National Institute for Health and Care Excellence adoption requires substantial, expert pathology knowledge.

Pathologists supporting clinical trials in local hospitals, and leading the pathology for multicentre clinical trials, require knowledge of the design and governance of trials, familiarity with laboratory protocols and an understanding of the analytical and biobanking aspects of trial management. This has relevance for both trials requiring patient stratification (close to a diagnostic activity) and those aiming to discover new determinants of response.

The management of pathological information in the context of LIMS (laboratory information management systems) and its integration in the context of hospital information systems will improve the quality of the diagnostic service. Equally, the use of this information for research purposes, within the requirements of anonymity, confidentiality and ethical approval, will facilitate hospital-based discovery.

### Clinical and scientific innovation

The development of digital pathology, pathology bioinformatics and biobanking represent critical areas of pathology activity in scientific discovery. Digital pathology and the adoption of image analysis have grown rapidly in the last few years due to advances in software, hardware and computer processing capacity, as well as the increasing importance of tissue-based research for biomarker discovery and stratified medicine. Digital pathology and image analysis have important roles across the drug/companion diagnostic development pipeline and in molecular pathology studies, tissue microarray (TMA) analysis, biobanking and molecular profiling of tissue. Integrating digital image analysis data with epidemiological, clinical and genomic data will help maximise the potential of tissue samples in delivering personalised medicine.^23^

The analysis and management of large genomics data will be at the heart of the delivery of the promise of modern genomic medicine. Effective integration of morphology with the ample available genomic information will determine the value of pathology in the future.

Repositories containing high-quality human biospecimens linked with robust and relevant clinical and pathological information are required for the discovery and validation of biomarkers for disease diagnosis, progression and response to treatment.^24^ It is imperative that modern biobanks provide the samples needed for discovery projects and ensure requirements for ongoing sample collections and the future needs of researchers are adequately addressed. Biobanks can work with molecular diagnostic laboratories to develop standardised methodologies for the acquisition and storage of samples required for new approaches to research such as ‘liquid biopsies’, which will ultimately feed into validations required in large prospective clinical studies in order to implement liquid biopsy approaches for routine clinical practice. This will need to be managed by pathologists understanding a large variety of technical aspects, ranging from traditional histopathology to the specific work of formalin-fixed paraffin-embedded-based DNA and RNA testing.

### Understanding disease

Both Pathology and Epidemiology aim to elucidate the aetiology of disease and the integrated discipline of ‘molecular pathological epidemiology’ aims to interrogate large sample cohorts from epidemiological studies with pathological tools such as IHC and TMAs, digital image analysis and high-throughput genomics, and link with epidemiological data to reach significant and practical discoveries.

In recent decades, pathology has witnessed a separation of the academic and diagnostic, to the extent that many Pathology departments in leading UK teaching hospitals lack any substantial academic activity. Activity to realign the diagnostic and research agendas, with specific models to do so, is gaining traction in the UK. The aim is to draw technological expertise from the research environment and to bring a culture of accreditation and rigour from diagnostics into research.

## Models of training from a UK perspective

The aim of this document is to propose key points of considerations for training the next generation of pathologists and pathology leaders, equipped to deliver healthcare founded on morphomolecular diagnoses. But how much molecular pathology should a diagnostic pathologist know?

It is a common opinion that the diagnostic histopathologist should integrate morphological and molecular information. There are two types of integration to consider: diagnostic and therapeutic. Diagnostic integration is exemplified by the haemato-oncology integrated report, where the result of clonality testing or the presence of a translocation can confirm/refute a diagnosis of malignancy and provide a clear diagnostic certainty. This paradigm is shared by, at least, neuropathology and sarcoma/soft tissue pathology.

Therapeutic integration informs a therapeutic decision. This requires very broad knowledge and specific training in molecular diagnostics. It requires:

- an understanding of the technologies that generated the results;
- judgement as to whether a specific test is technically satisfactory with the ability to troubleshoot a suboptimal test run;
- an understanding of the biological variables that some of these tests may bring to the forefront in some cases;
- knowledge to manage increasingly complex data (ie, bioinformatics);
- an understanding of the clinical relevance of test results.

At the moment, pathology training does not deliver these key skills and recent attempts to redefine training, while admirable, remain too limited. Training programmes that can produce pathologists who are also proficient in molecular diagnostics need to be developed, although there are significant challenges in developing such a programme; training as a competent morphologist in most areas of pathology requires a full 5 years of training, there are very few molecular pathology centres with the capacity to train a steady stream of molecular pathologists and the process for modifying a curriculum through the General Medical Council (GMC) is complex and lengthy. Here, we consider three potential models of delivering a future pathology workforce in the UK, taking as a baseline the current training structured championed by the Royal College of Pathologists.

### Model 1

Model 1 would aim to deliver morphomolecular training within the current 5-year training timeframe. This would require some condensation of the current morphological training, perhaps by allowing autopsy or gynae cytopathology to be dropped after stage A of training, and delivering a select amount of molecular training. This would include molecular pathology lectures in the early stages of training, followed by a compulsory 2-month to 3-month molecular attachment and an optional final year training in molecular diagnostics. Overall, this represents a meaningful increase in the molecular diagnostic training within the existing timeframe and structure of general histopathology training. Early training in the module has the capacity to be delivered in national/regional blocks, and there is the potential for a distance learning component in the pre-FRCPath Part 1 phase. This increase in molecular diagnostic training would need to be matched by an equally significant examined component of the FRCPath examinations. This model has the advantage of delivering a basic level of molecular pathology training to all, with a view to delivering an entire workforce with background in molecular diagnostics and the flexibility in stage D for suitably interested trainees to develop these skills further. Delivering this model of training within the currently existing 5-year RCPath histopathology training can be done in the appropriate centre,^25^ but nationwide coverage of molecular diagnostics may be insufficiently uniform at present to deliver the required level of practical molecular pathology training within each UK training region.

### Model 2

Model 2 focuses the delivery of molecular pathology training within an optional extension of training to allow an MSc or Fellowship in Molecular Pathology to be undertaken before or after the FRCPath Part 2. A plethora of Molecular Pathology MScs are currently available in the UK through which this additional training could be delivered to the relevant trainees. As the MSc programmes are optional, this could be perceived as an additional burden beyond the already demanding requirements of histopathology training, however, and if undersubscribed by pathology trainees, molecular pathology MSc programmes may fail to address the skills gap in molecular pathology. It is crucial that if these programmes aim to upskill trainees in molecular diagnostic pathology, the projects offered allow development of expertise in this area and are not predominantly focused on delivering basic science projects, which are used primarily as a segue into academic training.

### Model 3

Model 3 would involve targeted centres, which show they have the capacity to train morphomolecular pathologists, to start doing so, in programmes accredited by the RCPath, in parallel to conventional histopathology training. This would be delivered within the context of a modular training programme combining morphological and molecular training and would follow an approved RCPath template. It is envisaged that while this ‘hybrid’ morphomolecular training programme is delivered in selected centres, the majority would continue with the standard training currently being delivered to histopathology trainees. These parallel training programmes would potentially reflect more accurately the true distinction between general pathologists, who require the balanced general training to report a wide range of specimens, completing training the current RCPath curriculum (or proposed model 1), and pathologists who practice monospecialist reporting in centres that also deliver molecular pathology testing being trained through model 3.

Molecular pathology is a vast area that is constantly evolving, and it is difficult to have a grasp of every technique and application and to learn about techniques in an abstract manner. It would therefore be useful to link molecular techniques with the context of a pathology subspecialty within a modular training programme. This proposal will prove some logistical challenges to the relevant training centres and the RCPath, although the model would not be unlike the specialist training delivered and examined after stage B in head and neck pathology for dentally qualified pathologists.

Due to the national strategic importance of genomic medicine within the UK and the already problematic skills gap in molecular pathology, such morphomolecular training posts should be considered additional to the existing histopathology training numbers with a national-level funding model that reflects this.

## Histopathology in 2017: a time to exercise responsibility

The discipline of histopathology is at the crossroads. To maintain its central role in the evaluation of patients, pathologists will need to fully incorporate molecular diagnostics for the understanding and characterisation of diseases. If surgical pathologists attempt, even passively, to resist this change, this is likely to invite others to perform molecular testing of surgical pathology samples, moving the central role of tissue-based diagnosis out of the field of pathology. Crucially, this will be a poor outcome for patients, as morphological and molecular knowledge that needs to be integrated will be fragmented into different specialities. The strategic position of our discipline at the centre of clinical practice may be lost, reducing the importance of histopathology as a clinical specialty.

The resulting marginalisation of histopathology in molecular medicine will further diminish the current strategic position of histopathology at the interface between clinical medicine and biomedical research. Failure to learn the language of molecular biology will significantly reduce the input and engagement from histopathologists in tissue-based research and their valuable skills will become a more remote resource to the biomedical research community, which will ultimately impact negatively on the quality of research. In addition, molecular diagnostics is a rapidly growing area of medicine, moving a budget of many billions of dollars, and appropriately trained molecular pathologists are potentially best placed to make appropriate decisions about how these considerable investments are made to support optimal, combined morphomolecular diagnostics. Histopathologists’ disengagement from molecular medicine threatens to exclude our departments from significant potential sources of revenue.

The CM-Path group authoring this document has appraised the options identified for training the next generation of histopathologists. The group believes that molecular diagnostic training should represent a substantial component of the histopathology curriculum in the UK, far beyond what is currently delivered, although it is understood that the Royal College of Pathologists’ position is that there is little margin to change the curriculum at this time.

A revisited version of today’s curriculum, with a significant increase in molecular diagnostic training, taking place within the current 5-year programme (described in model 1) and increasing the time devoted to the theory and practice of molecular pathology is certainly the minimum change that the group feels is required to properly equip the specialty for future pathology practice. Model 2 of training has potential as an interim solution while more radical change in the curriculum is developed although the optional and variable nature of the molecular pathology component makes this an inadequate long-term plan for upskilling the profession.

Overall, the CM-Path group considers that, in order to secure the future of the specialty at the fulcrum of modern medicine, a parallel training programme should be implemented. This would include one pathway based on current training, which it is envisaged the vast majority will follow in the first instance, and an alternate option of incorporating in-depth molecular diagnostic training over the 5 years by developing modular morphomolecular pathology training programmes (model 3). This parallel subspecialist pathway will develop a cohort of highly skilled molecular histopathologists both able to strategically secure tissue-based molecular diagnostics within our specialty and capable of embracing the inevitable increase in morphomolecular diagnostic complexity that each subspecialty will face over the coming years.

It is our view that maintaining the status quo will be detrimental for both the next generation of pathologists and for the specialty as a whole. Whether or not we embrace radical change, the eventual future of histopathology will be our responsibility. Do we need to nurture molecular pathology as much as morphology? How much of the revolution in molecular medicine do we wish to embrace? These are questions that our generation can ask itself, though if we do not act accordingly, we may be the last generation of pathologists able to do so.

**Take home messages**

- Molecular diagnostics is one of the fastest growing areas of medicine.
- The involvement of pathology in the modern taxonomy of disease has become a matter of general importance for the medical and scientific community.
- Pathology and pathologists need to rise to the challenge of genomic medicine, this will dictate the extent and quality of genomic medicine over the next decade.
- The National Cancer Research Institute’s CM-Path initiative would like to see a change in the postgraduate pathology curriculum, to create a new morphomolecular pathology specialty in order to deliver high quality molecular diagnostics for patient benefit.
